# A Mechanism‐Based Framework for Anti‐Aging Strategies: From Metabolic Regulation to Senotherapy and Stem Cell‐Based Interventions

**DOI:** 10.1111/acel.70658

**Published:** 2026-08-27

**Authors:** Feifei Li, Yankai Wang, Gelin Wang, Jiguo Chen, Song Huang

**Affiliations:** ^1^ National Institute of Biological Sciences Beijing China; ^2^ Medical AI Technologies & Robotics Institute Xiong'an China; ^3^ Institute for Translational Medicine on Cell Fate and Disease, Shanghai Ninth People's Hospital, Key Laboratory of Cell Differentiation and Apoptosis of National Ministry of Education, Department of Pathophysiology Shanghai Jiao Tong University School of Medicine Shanghai China; ^4^ Tsinghua Institute of Multidisciplinary Biomedical Research Tsinghua University Beijing China

**Keywords:** age‐related diseases, aging, anti‐inflammaging, metabolic modulation, senotherapy

## Abstract

The global prevalence of aging and age‐related diseases has increased markedly in recent decades due to extended life expectancy and a growing aging population, posing substantial medical and social burdens. Multiple strategies, including metabolic modulation (e.g., physical exercise, calorie restriction, and calorie restriction mimetics), targeting inflammaging, senotherapy, parabiosis, stem cell‐based therapies, and epigenetic rejuvenation, have shown promise in slowing aging and extending lifespan in preclinical models, with some demonstrating efficacy in clinical trials. This review summarizes the current status of leading anti‐aging interventions, their clinical progress, and the underlying mechanisms, which include enhancing autophagy, clearing senescent cells and supporting mitochondrial function to reduce chronic inflammation. We propose that metabolic modulation, inflammaging control, and senotherapy constitute three interconnected pillars of contemporary anti‐aging strategies.

## Graphical abstract

The aging of the human body is a complex process driven by multiple contributing factors. Based on their underlying mechanisms, current anti‐aging interventions can be classified into six primary categories. This paper summarizes the most promising anti‐aging strategies within each class.

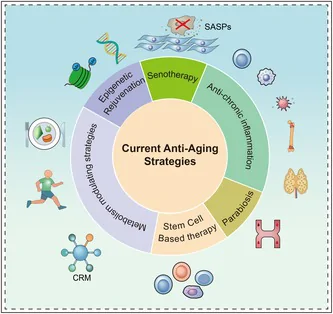

## 1 Introduction

Global population is aging rapidly. According to the United Nations' World Population Prospects 2019 (United Nations et al. 2019), one in six individuals worldwide will be over 65 by 2050. Aging is characterized by a gradual and progressive decline in physiological function, which markedly increases the risk of chronic diseases, including cardiovascular diseases, cancer, diabetes, and neurodegenerative disorders. Over the past century, advancements in food supply, healthcare, and infectious disease control have substantially extended human lifespans. However, this increased longevity has been accompanied by a sharp rise in age‐related morbidity. As the aging population grows, the social, economic, and healthcare burden are escalating, underscoring an urgent need for strategies that delay age‐associated diseases and promote healthy aging.

Over the past two decades, the biology of aging has garnered significant research interest, grounded in the understanding that while aging itself is inevitable, its rate, trajectory, and pathological consequences are highly plastic and malleable, while many age‐related diseases are preventable or delayable. In 2000, Holloszy proposed that aging can be distinguished between disease‐independent primary aging and disease‐accelerated secondary aging, noting that factors influencing longevity primarily affect disease development, thereby improving secondary aging (Holloszy 2000). He stated that aging was driven by the loss of homeostasis through three systems—nutrient sensing, hormonal function, and immune response.

In 2013, López et al. provided a unifying framework to describe the fundamental biological processes of aging by outlining nine hallmarks of aging (López‐Otín et al. 2013). These hallmarks were expanded to 12 in 2023, and to 14 in 2025 (López‐Otín et al. 2023; Kroemer et al. 2025). These hallmarks are highly interconnected yet heterogeneous. They span multiple biological scales, ranging from molecular changes (e.g., genomic instability, telomere attrition) to cellular dysfunctions (e.g., loss of proteostasis, mitochondrial dysfunction) and tissue‐ or systemic‐level alterations (e.g., stem cell exhaustion, chronic inflammation, and dysbiosis). They also differ in causal roles, with some acting as upstream drivers of aging and others representing downstream consequences or phenotypic manifestations.

**FIGURE 1:**
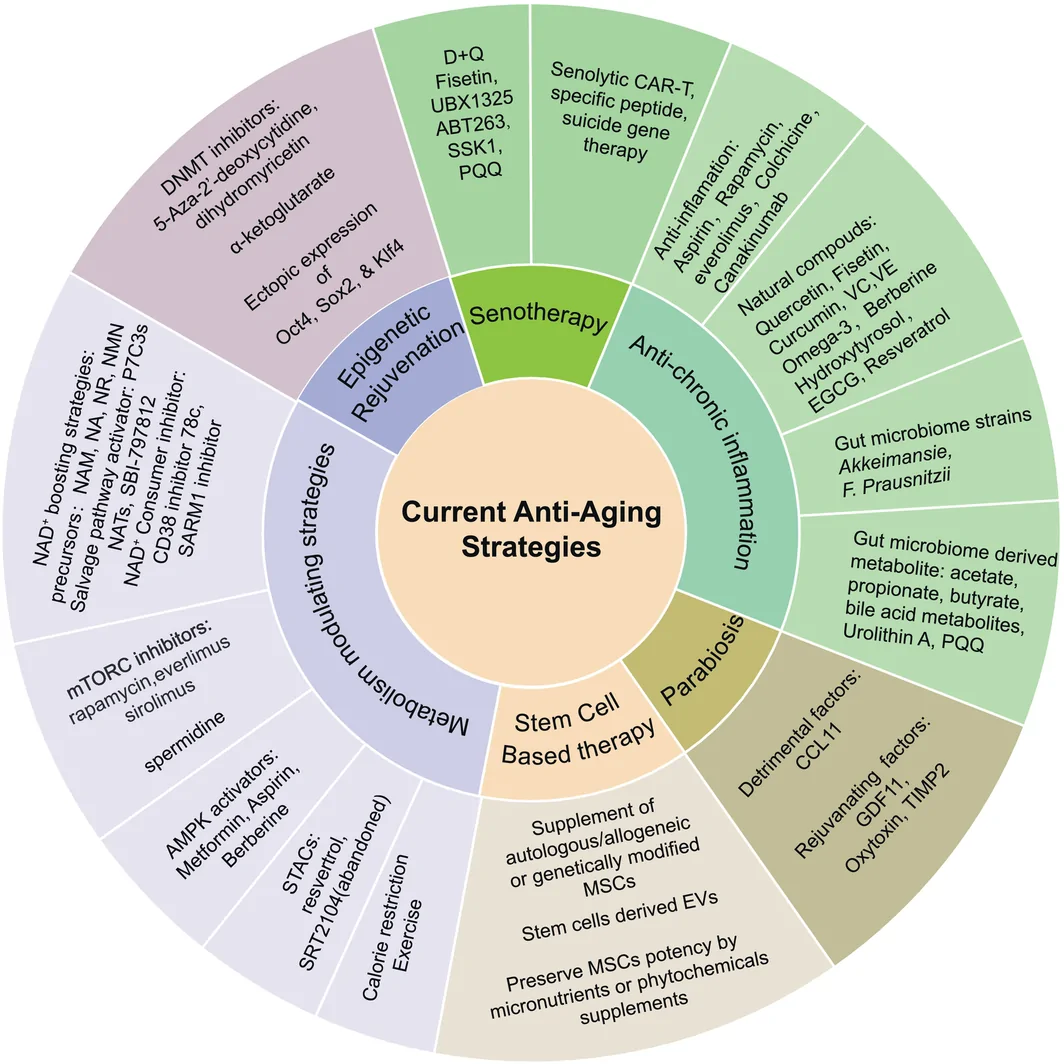
Summary of major current anti‐aging strategies. The main categories of anti‐aging interventions include metabolism modulation, senotherapy, suppression of chronic inflammation, stem cell supplementation, parabiosis, and epigenetic rejuvenation. Detailed descriptions of each strategy are provided in the main text.

In parallel with these conceptual advances, a growing number of interventions have been shown to extend lifespan and improve healthspan in preclinical models. However, existing anti‐aging strategies are often discussed in isolation, resulting in a fragmented view of the field. Here, we propose a mechanism‐based framework that organizes current anti‐aging strategies into six major categories according to their primary biological targets: metabolic regulation, control of chronic inflammation, senotherapy, parabiosis, stem cell–based therapies, and epigenetic rejuvenation (Figure 1). We summarize the mechanistic rationale underlying each category, review representative interfering approaches, and discuss their developmental status, limitations, and translational challenges. By categorizing diverse interventions according to their underlying mechanisms, this review aims to provide a structured and accessible overview of the current anti‐aging landscape while highlighting potential connections and interactions among different strategy classes.

## 2 Metabolism Modulating Strategies

**FIGURE 2:**
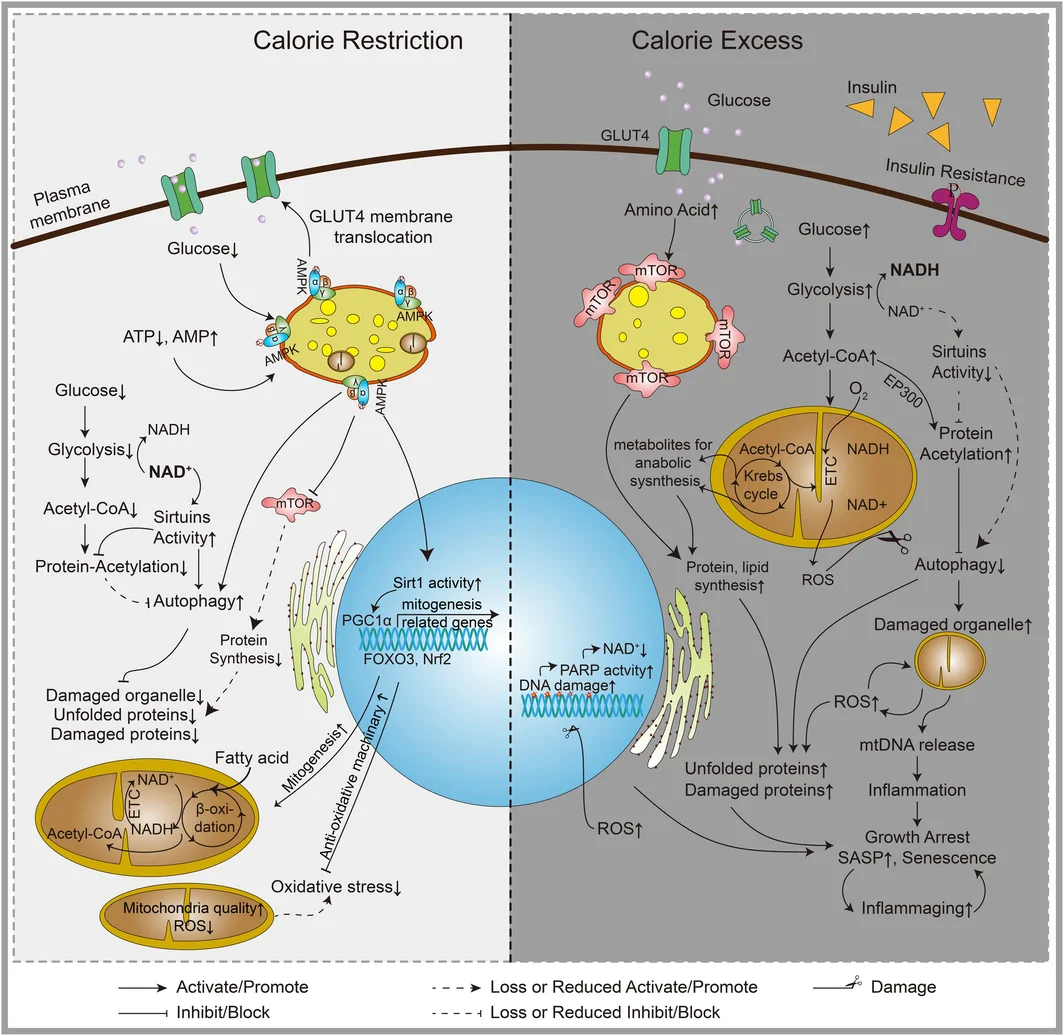
Conserved metabolic pathways underlying anti‐aging interventions. Evolutionarily conserved metabolic pathways—insulin/IGF‐1 signaling (IIS), mTORC1, AMPK, and sirtuins—serve as central targets for anti‐aging interventions. Under nutrient‐rich conditions, insulin activates the IIS pathway, promoting GLUT4 translocation to the plasma membrane and increasing glucose uptake. IIS activation and amino acid availability stimulate mTORC1, enhancing anabolic metabolism to support cellular growth and proliferation. In this state, intracellular acetyl‐CoA levels and global protein acetylation are elevated, while autophagy is suppressed. During aging, sustained mTORC1 activation coupled with impaired autophagy leads to the accumulation of damaged proteins and organelles, triggering cellular stress responses and senescence, and contributing to inflammaging. The resulting pro‐inflammatory milieu further exacerbates metabolic dysfunction by inducing insulin resistance through inhibitory phosphorylation of the insulin receptor. Conversely, activation of AMPK—triggered by calorie restriction, physical exercise, or pharmacological agents such as metformin—restores metabolic homeostasis through multiple mechanisms. AMPK promotes glucose uptake via GLUT4 translocation independently of IIS, enhances stress resistance through FOXO and NRF2 signaling, supports mitochondrial biogenesis via PGC‐1α, and increases autophagy. Simultaneously, AMPK restrains excessive anabolic metabolism by inhibiting mTORC1. Autophagy is differentially regulated by nutritional status. A global reduction in protein acetylation, reflecting depleted acetyl‐CoA levels and low energy status, robustly induces autophagy. Concurrently, SIRT1‐mediated deacetylation of ATG5, 7, and 8 is essential for autophagy initiation. NAD^+^ metabolism and sirtuin signaling are integral to metabolic regulation during aging. NAD^+^ levels and SIRT1 activity directly modulate autophagy via acetylation. An age‐related decline in NAD^+^ diminishes SIRT1 activity, leading to the hyperacetylation of the NF‐κB p65 subunit; this sustains its nuclear activity and drives the transcription of SASP factors. Furthermore, depleted NAD^+^ levels induce mitochondrial dysfunction and mtDNA leakage into the cytosol, thereby triggering the cGAS‐STING pathway and SASP overexpression. Ultimately, impaired SIRT1 activity, coupled with accumulated DNA damage, results in p53 hyperacetylation, irreversible cell cycle arrest, and senescence.

The first evidence linking metabolism to aging was reported by McCay and colleagues in 1935, demonstrating that a 30%–40% reduction in energy intake prolonged lifespan in rats (McCay et al. 1935). Also, the first longevity‐associated gene identified, AGE‐1, encodes a PI3K homolog in *Caenorhabditis elegans* that plays a fundamental role in energy metabolism and growth (Friedman and Johnson 1988; Johnson 1990). Mutation of daf‐2, the insulin/insulin‐like growth factor signaling (IIS) receptor, can extend *C. elegans* lifespan by two‐ to threefold (Kenyon et al. 1993). Pharmacological inhibition of intestinal glucose absorption with acarbose also exhibits anti‐aging effects and extends lifespan in mice (Harrison et al. 2014; Harrison et al. 2019). These findings established that evolutionarily conserved metabolic pathways are critical regulators of aging (see Figure 2). Dysregulation these metabolic pathways are frequently observed in aging and disease (Smith et al. 2008; Barzilai et al. 2012), and genetic or pharmacological manipulation of these pathways has been shown to influence lifespan and healthspan in model organisms (Barzilai et al. 2012; Barzilai et al. 2016).

### 2.1 Metabolism Pathways in Aging

The phosphatidylinositol kinase‐related kinase family member mTOR integrates nutrient and stress signals to regulate cell growth and survival. Studies in *Saccharomyces cerevisiae* (budding yeast), *Drosophila melanogaster* (fruit fly), and *C. elegans* have identified the mTOR pathway as a negative regulator of lifespan (Papadopoli et al. 2019). Aberrant mTOR activation is associated with multiple aging hallmarks, including impaired nutrient sensing, loss of proteostasis, defective autophagy, mitochondrial dysfunction, cellular senescence, and stem‐cell exhaustion. Dysregulated mTOR activity is also observed in age‐related diseases such as type 2 diabetes, cancer, cardiovascular disease, and several neurodegenerative disorders. Inhibition of mTOR, either genetically or pharmacologically, extends lifespan in yeast, worms, flies, and mice (Um et al. 2004; Selman et al. 2008; Harrison et al. 2009; Kapahi et al. 2010).

In contrast, AMPK serves as the master cellular energy sensor, activated under low‐energy conditions (Hardie et al. 2012; Lin and Hardie 2018). Classically, AMPK activation is triggered by an increased AMP/ATP ratio, reflecting energy stress, or by elevated intracellular Ca^2+^ (Hardie et al. 2012). More recently, a lysosome‐associated AMPK activation pathway has been identified, independent of the AMP/ATP ratio and regulated primarily by glucose availability and v‐ATPase activity (Zhang et al. 2014; Zhang et al. 2017; Lin and Hardie 2018). Upon activation, AMPK reduces anabolic processes and enhances catabolic pathways to restore energy balance (Burkewitz et al. 2014). However, AMPK responsiveness declines with age (Reznick et al. 2007; Ljubicic and Hood 2009). Activation of AMPK has been shown to confer multiple anti‐aging benefits. At the molecular level, AMPK promotes autophagy by inhibiting mTOR and phosphorylating ULK1 (Kim et al. 2011; Salminen and Kaarniranta 2012), enhances mitochondrial fatty acid β‐oxidation, increases cellular NAD^+^ levels, activates SIRT1, and induces PGC‐1α to stimulate mitochondrial biogenesis (Cantó et al. 2009). Moreover, AMPK activation modulates age‐related immune dysregulation, partly via NF‐κB inhibition (Salminen et al. 2011). Collectively, AMPK is a central target in multiple anti‐aging interventions, including CR, exercise, and pharmacological agents.

Sirtuins are a family of NAD^+^‐dependent deacetylases and mono‐ADP‐ribosyltransferases that links metabolism to aging (Houtkooper et al. 2012). The discovery that Sir2 overexpression extends lifespan sparked widespread interest in sirtuins (Kaeberlein et al. 1999). Mammals possess seven sirtuins (SIRT1–7), localized to distinct subcellular compartments and involved in diverse biological processes (Houtkooper et al. 2012). Sirtuin activation, particularly of SIRT1, has been observed under CR, exercise, and other anti‐aging interventions (Lin et al. 2000; Cantó et al. 2009). Strongly regulated by NAD^+^ availability and the NAD^+^/NADH ratio, sirtuins coordinate cellular responses to energy status and stress by deacetylating a broad range of targets. For instance, SIRT1‐mediated deacetylation of PGC‐1α promotes mitochondrial biogenesis (Rodgers et al. 2005), while deacetylation of FOXO1 and FOXO3 regulates glucose and lipid metabolism (Lee and Dong 2017). SIRT3, localized to mitochondria, enhances fatty acid β‐oxidation by activating long‐chain acyl‐CoA dehydrogenase (Hirschey et al. 2010). SIRT1 also suppresses inflammation by deacetylating NF‐κB (Lee and Dong 2017). Sirtuin activation is protective against age‐related metabolic disorders such as diabetes and obesity, as well as neurodegenerative diseases like AD and PD. Small‐molecule sirtuin activators (STACs) represent a major class of candidate anti‐aging agents (Hubbard and Sinclair 2014).

These metabolic pathways form a highly interconnected regulatory network, translating diverse metabolic states into distinct aging phenotypes. Distinct modulation strategies target different entry points within this network, suggesting that combinatorial approaches may yield additive or synergistic anti‐aging effects (Kitaoka et al. 2016; Acosta‐Rodríguez et al. 2022; Ham et al. 2022).

### 2.2 Calorie Restriction (CR)

Clinical studies of CR to date have been largely positive. The phase I Comprehensive Assessment of Long‐term Effects of Reducing Intake of Energy (CALERIE) trial (2002–2004) demonstrated that 6 months of CR reduced fasting insulin levels and core body temperature, both considered biomarkers of longevity (Heilbronn et al. 2006). The phase II CALERIE trial (2007–2011) showed significant improvements in multiple cardiometabolic risk factors after a 2‐year, 25% reduction in caloric intake in healthy, non‐obese, middle‐aged adults (Kraus et al. 2019). A follow‐up observational study is currently underway to evaluate whether these benefits persist 10–15 years after CR (NCT05651620). Another study in obese older adults (> 65 years) at high cardiometabolic risk found that 54 weeks of CR did not significantly reduce visceral adipose tissue, but significantly decreased total body fat and cardiometabolic risk factors without notable loss of lean mass (Ard et al. 2016). CALERIE‐2 study further examined potential adverse effects of CR on mood, quality of life, sleep, and sexual function in healthy, non‐obese adults. Self‐reported outcomes showed that 2 years of 25% CR did not negatively affect health‐related quality of life (Martin et al. 2016). In addition to continuous CR, growing evidence supports the benefits of intermittent fasting and time‐restricted feeding (Morales‐Suarez‐Varela et al. 2021). However, longer‐term trials are required to determine whether CR can truly attenuate human aging.

The beneficial effects of CR are thought to be mediated through multiple aging‐related biological processes and pathways, including activation of AMPK (Cantó and Auwerx 2011), activation of sirtuins (Lin et al. 2000; Someya et al. 2010), inhibition of IIS and mTOR signaling (Heilbronn et al. 2006; Lee and Min 2013; Yang et al. 2014; Mitchell et al. 2015), reduction in AGEs levels (Cai et al. 2007; Chaudhuri et al. 2018), reduced adiposity, and elevated adiponectin levels (Fontana and Klein 2007; Miller et al. 2017). In addition, CR also reduces cytokine production and inhibits systemic inflammation, through reducing AGEs levels (Davis et al. 2016; Vella et al. 2023), as well as through modulation of gut microbiota (Lee and Longo 2011; Gabandé‐Rodríguez et al. 2019; Hernández‐Saavedra et al. 2019).

Physical exercise is also an extensively validated anti‐aging intervention, with benefits across multiple physiological systems, including skeletal muscle, the cardiovascular and respiratory systems, the nervous system, and whole‐body metabolism. Exercise has even been proposed to counteract aging by modulating nearly all recognized hallmarks of aging (Rebelo‐Marques et al. 2018).

### 2.3 Calorie Restriction Mimetics (CRMs)

CR and physical exercise are widely regarded as “polypills” for the prevention of multiple age‐related diseases. However, both require sustained effort and strict adherence, which limits long‐term compliance and broad applicability. Consequently, there is strong interest in identifying pharmacological agents and dietary supplements capable of mimicking the effects of CR and exercise. Among the most widely investigated CRMs are AMPK activators, mTOR inhibitors, as well as autophagy inducers (Table 1).

**TABLE 1:** Metabolism modulating strategies.

Compounds	Targets/cellular process	Current status	Origin
Metformin	Multi‐targets: AMPK, Complex I of ETC, GPD2	Improve cognitive impairment in frail older women with hypertension and diabetes (Mone et al. 2023), ANTHEM clinical trial ongoing to reveal whether metformin is harmful to insulin‐sensitive healthy individuals (Kumari et al. 2021) Large scale randomized clinical trial to support its anti‐aging effects in non‐diabetic individuals planned (TAME)	Natural Products
Rapamycin (Sirolimus)	mTOR	Extend lifespan in model organisms (Harrison et al. 2009). Low‐dose (10 mg/week), intermittent rapamycin administration for 48 weeks is safe improved lean tissue mass in women (Moel et al. 2025) Undergoing a phase IIa clinical trial for its impact on aging treatment and cerebral glucose uptake in early‐stage AD (NCT06022068) (Svensson et al. 2024)	Natural products from bacteria
Everolimus	mTOR	Improve immune function in aged volunteers, Small scale clinical trial (Mannick et al. 2014) Under clinical evaluation for efficacy in prevent bone loss in postmenopausal women (NCT06789900)	Synthetic
Resveratrol	SIRT1	About 200 clinical trials without a clear‐cut conclusion. Reported beneficial effects include improvement of cardiovascular health, muscle structure and function insulin sensitivity and dysregulated metabolism, as well as reduced inflammation. No significant improvement in these aspects was also reported in other studies (Toniolo et al. 2023; Brown et al. 2024)	Natural products from grapes, red wine etc.
SRT2104	SIRT1	Phase II clinical trial failed to show efficacy toward type II diabetes (Baksi et al. 2014), further development discontinued	Synthetic
Acarbose	Inhibit α glucosidase to block glucose absorption	Preclinically proved to extend mice lifespan (Harrison et al. 2014; Harrison et al. 2019). Clinical trials conducted to evaluate acarbose anti‐aging effects (NCT02865499); another phase II study (NCT02953093) proved Acarbose altered gene expression of pathways related with aging in muscle and adipose tissues	Natural products from bacteria
Aspirin and its metabolite salicylate	Multi‐target: AMPK, EP300, COX‐1	Negative clinical outcomes: Low‐dose aspirin failed to prove positive effect in healthy aging in ASPREE clinical trial (McNeil, Nelson, et al. 2018; McNeil, Wolfe, et al. 2018; McNeil, Woods, et al. 2018). Aspirin decreased the rate of serious vascular event in diabetic patients, but caused major bleeding events and failed to improve cognitive function in ASCEND clinical trial (Bowman et al. 2018; Parish et al. 2022). ARRIVE trial also failed to show beneficial effects (Gaziano et al. 2018)	Natural products
Spermidine	EP300	Extend lifespan in model organisms. Clinical trials: spermidine supplement was safely tolerated in older adults (Schwarz et al. 2018), cannot increase spermidine levels in blood (Senekowitsch et al. 2023), no improvement on memory performance in older adults (Schwarz et al. 2022). Safety profile and efficacy on glucose homeostasis in prediabetic individuals with elevated blood glucose levels are under clinical evaluation (NCT06445569)	Natural metabolites
Nicotinamide riboside	Boost NAD^+^ levels	Preclinical proved NR supplement failed to improve skeletal muscle function, exercise capacity and/or insulin sensitivity in clinical trials. Clinical study is underway to evaluate whether exercise can elicit the benefit effect of NR supplement (NCT06425042, NCT04907110). Phase II clinical study for the potential of NR supplement to decelerate functional decline in elderly frail population (NCT06208527) Phase 4 study on NR effect to improve brain health and memory (NCT05483465)	
NMN	NAD^+^ precursors	Under clinical evaluation for safety and efficacy in improving exercise tolerance in healthy older adults (NCT07144527)	

#### 2.3.1 Metformin and Berberine

Metformin has been safely used for nearly 70 years as a first‐line treatment for type 2 diabetes, during which its pleiotropic effects—including reduced appetite, lower circulating free fatty acid, and weight loss—have attracted considerable attention. Preclinical studies have reported lifespan extension by metformin in multiple animal models, including nematodes, mice, and monkeys (Anisimov et al. 2005; Martin‐Montalvo et al. 2013; Yang et al. 2024). Accumulating evidence also supports a role for metformin in mitigating several hallmarks of aging (Kulkarni et al. 2020).

Whether metformin can be safely repurposed as an anti‐aging intervention is under rigorous clinical investigation. In a small‐scale trial in older individuals with impaired glucose tolerance, 6 weeks of metformin not only modulated pathways related to glucose metabolism and mitochondrial β‐oxidation but also affected DNA repair in muscle and collagen trimerization in adipose tissue (Kulkarni et al. 2018). In frail older women with hypertension and diabetes, 6 months of extended‐release metformin significantly improved cognitive function (Mone et al. 2023). However, it remains unclear whether metformin benefits non‐diabetic individuals and whether specific criteria can identify those most likely to respond. The ongoing Antecedent Metabolic Health and Metformin study is designed to assess whether metformin may be detrimental in healthy insulin‐sensitive individuals (Kumari et al. 2021). Another landmark study, the Targeting Aging with Metformin trial, is designed to test whether metformin can delay the onset or progression of age‐related diseases—including neurodegenerative disorders, cardiovascular disease, and cancer—in non‐diabetic individuals aged 65–79. This six‐year study plans to recruit 3000 participants across 14 U.S. sites, with the primary goal of establishing proof‐of‐concept that aging itself can be targeted as a treatable indication.

Metformin exerts its pleiotropic effects through multiple mechanisms and targets. Reported targets contributing to its anti‐aging effects include mitochondrial complex I of the ETC (Reczek et al. 2024), mitochondrial glycerophosphate dehydrogenase (mGPD) (Madiraju et al. 2014), and PEN2 in the v‐ATPase–AMPK axis (Ma et al. 2022).

Traditionally employed to treat infectious diarrhea and dysentery, berberine has emerged in preclinical studies as a potent multifunctional metabolic modulator. Its diverse activities encompass the activation of AMPK signaling (Yin et al. 2008), enhancement of insulin sensitivity (Bustanji et al. 2006), and optimization of gut microbiota composition, lipid metabolism, and the adipokine network (Zieniuk and Pawełkowicz 2025). Future clinical research is warranted to validate these findings in humans and translate berberine into robust anti‐aging therapeutic strategies.

#### 2.3.2 Resveratrol and Sirtuin‐Activating Compounds (STACs)

Resveratrol is the first‐generation SIRT1 activator. The “French Paradox” linking red wine consumption to lower cardiovascular disease rates popularized resveratrol as a compound with potential health benefits. Preclinical studies demonstrated that resveratrol can extend lifespan in model organisms (Valenzano et al. 2006; Yu and Li 2012; Bhullar and Hubbard 2015). Resveratrol activates SIRT1, which induces PGC1α expression to improve mitochondrial function and increase oxygen consumption in mice (Lagouge et al. 2006), supporting its classification as a CRM (Chung et al. 2012).

Clinical outcomes of resveratrol have been mixed: some trials report improvements in metabolic profiles, cardiovascular health, insulin sensitivity, and reduced inflammation, while others show no significant effects (Timmers et al. 2011; Toniolo et al. 2023; Brown et al. 2024; Godos et al. 2024). Poor bioavailability, dosage variations, study design, comorbidities, lifestyle factors and interactions with diverse gut microbiota likely contribute to these inconsistencies (Cottart et al. 2010). Efforts to enhance resveratrol stability and bioavailability, such as through colloidal carriers, are ongoing (Abbas et al. 2018).

Beyond resveratrol, a wide range of synthetic STACs have been developed and tested for potential anti‐aging effects. To date, only one candidate, SRT2104, progressed to clinical trials but failed to demonstrate significant efficacy in patients with type 2 diabetes (Baksi et al. 2014). Given that sirtuin activity is tightly dependent on intracellular NAD^+^ availability, current strategies are exploring combinations of STACs with NAD^+^‐boosting interventions in an effort to enhance their therapeutic efficacy (Imai and Guarente 2014).

#### 2.3.3 Rapamycin and Rapalogs

Rapamycin was discovered in the mid‐20th century as an antifungal from soil bacteria (Arriola Apelo and Lamming 2016), and gave its name to its molecular target, the mammalian target of rapamycin (mTOR). Rapamycin was approved by the FDA in 1999 as an immunosuppressant for kidney transplant rejection, and its later‐recognized anti‐proliferative activity led to the development of rapalogs such as everolimus, temsirolimus, and ridaforolimus for cancer therapy. Given the central role of mTOR in metabolism and aging (Johnson et al. 2013), rapamycin and its analogs have been extensively studied as geroprotective agents. Landmark findings in 2009 demonstrated that rapamycin extends lifespan in mice (Harrison et al. 2009), and subsequent studies have consistently supported lifespan extension through mTORC1 inhibition in diverse model organisms, including yeast, worms, fruit flies, and mice (Harrison et al. 2009; Johnson et al. 2013; Kennedy and Lamming 2016; Papadopoli et al. 2019).

Several clinical evaluations are underway to assess their anti‐aging potentials. In a pioneering trial, everolimus enhanced immune function in elderly volunteers (Mannick et al. 2014). The PEARL trial demonstrated that low‐dose, intermittent rapamycin (10 mg/week for 48 weeks) was safe and increased lean tissue mass in older women (Moel et al. 2025). More trials are ongoing. A phase IIa study is testing whether rapamycin alters cerebral glucose uptake in early‐stage AD patients (Svensson et al. 2024). The RAPID trial is exploring rapamycin's ability to improve oral health in older adults, while everolimus is under evaluation for its potential to mitigate bone loss in postmenopausal women (NCT06789900). Meanwhile, pharmacokinetic (PK) and pharmacodynamic (PD) profiles of sirolimus and everolimus in older adults are currently under clinical evaluation (NCT06658093).

Common adverse effects of mTOR inhibitors include increased infection risk, insulin resistance, and impaired wound healing (Sankhala et al. 2009). For the long‐term use of mTOR inhibitors to delay aging and age‐related diseases, these side effects must be carefully managed. Strategies under investigation include developing more selective mTORC1 inhibitors to minimize mTORC2‐related adverse effects, optimizing intermittent dosing, and combining mTORC1 inhibitors with other caloric restriction mimetics, such as metformin, to improve safety and efficacy.

#### 2.3.4 Spermidine

Spermidine is a naturally occurring polyamine found in all living organisms. Its levels decline with age, but remain elevated in healthy nonagenarians and centenarians (Pucciarelli et al. 2012). It regulates diverse cellular processes and has pleiotropic effects, including anti‐inflammatory and antioxidant actions, improved mitochondrial function, and enhanced proteostasis (Madeo et al. 2018). Mechanistically, spermidine inhibits the cytosolic acetyltransferase EP300, reducing global protein acetylation and thereby promoting autophagy, which largely underlies its beneficial effects (Eisenberg et al. 2009; Pietrocola et al. 2015; Zhang et al. 2019; Zhang and Simon 2020). A recent study reported that CR elevates spermidine levels in yeast, flies, mice, and humans, which is essential for mediating the beneficial effects of CR (Hofer et al. 2024).

Several clinical trials have evaluated spermidine supplementation in aging and age‐related conditions. In a 90‐day pilot study, spermidine supplementation prolonged the anagen phase of hair follicles, indicating potential benefits against hair loss (Rinaldi et al. 2017). However, a 12‐month spermidine‐rich diet didn't improve memory in older adults with subjective cognitive decline (Schwarz et al. 2022), and high‐dose spermidine supplementation failed to raise plasma or salivary spermidine levels in healthy adults (Senekowitsch et al. 2023). These results highlight ongoing challenges with spermidine's bioavailability and pharmacokinetics. Despite this, spermidine remains promising due to its robust safety profile and autophagy‐inducing effects. Translating its lifespan‐extending effects from preclinical models into humans will require rigorous fundamental studies, particularly those addressing absorption, distribution, metabolism, and overall bioavailability.

### 2.4 NAD ^+^ Boosting Strategies

Nicotinamide adenine dinucleotide (NAD^+^) is a central metabolic cofactor and signaling molecule regulating numerous age‐related processes (Covarrubias et al. 2021). It acts both as a redox carrier in glycolysis and mitochondrial oxidative phosphorylation and as a substrate for enzymes—including sirtuins, PARPs, CD38/CD157, and SARM1—that modulate genomic stability, protein modifications, epigenetics, neurodegeneration, and immune function (Li et al. 2024). NAD^+^ levels decline with age, contributing to frailty and increased risk of age‐related diseases (Yoshino et al. 2018).

The role of NAD^+^ in longevity was first shown in yeast, where CR failed to extend lifespan in strains lacking sir2, an NAD^+^‐dependent deacetylase, or NPT1, a key enzyme in NAD^+^ biosynthesis (Lin et al. 2000). Subsequent studies confirmed that NAD^+^ augmentation can extend lifespan in organisms ranging from fly to mammals (Mouchiroud et al. 2013; Bonkowski and Sinclair 2016). Indeed, many metabolism‐modulating interventions—such as CR (Wei et al. 2023), exercise (Walzik et al. 2025), and AMPK activation (Cantó et al. 2009; Qu et al. 2024)—promote healthy aging at least partly by increasing NAD^+^ levels.

NAD^+^ precursors, including NA, NR, and NMN, are being investigated for age‐related diseases in preclinical and clinical studies (Li et al. 2024). Reported clinical benefits include increased NAD^+^ levels, telomere lengthening in peripheral blood mononuclear cells, reduced inflammatory cytokines, and improved muscle and physical function (Elhassan et al. 2019; Pirinen et al. 2020; Niu et al. 2021; Yi et al. 2023; Orr et al. 2024). However, NR did not improve cognition in older adults with mild cognitive impairment (Orr et al. 2024), and two long‐term trials found no cardiovascular benefit of NA, with significant gastrointestinal, musculoskeletal, and skin‐related adverse effects (Boden et al. 2011; Group 2013; Gasperi et al. 2019). Overall, evidence from large‐scale, long‐term trials remains insufficient.

Activating enzymes in the NAD^+^ biosynthetic pathway, particularly NAMPT, is another NAD^+^‐boosting strategy. As the rate‐limiting enzyme of the NAD^+^ salvage pathway, NAMPT controls the major source of cellular NAD^+^. NAMPT activators, including P7C3 and its analogues and NAT derivatives, increase NAD^+^ levels and protect against age‐related neurodegeneration in preclinical models (Yin et al. 2014; Bauman et al. 2018; Yao et al. 2022).

CD38 overexpression is a major driver of age‐related NAD^+^ decline (Camacho‐Pereira et al. 2016), making CD38 inhibition a promising anti‐aging strategy. The selective inhibitor 78c extended healthspan and lifespan in aged mice (Tarragó et al. 2018; Peclat et al. 2022; Tenchov et al. 2024), while the anti‐CD38 antibody daratumumab showed benefits in models of age‐related inflammation and metabolic dysfunction (Guerreiro et al. 2020). Natural compounds such as apigenin and quercetin also inhibit CD38 (Escande et al. 2013), and a CD38‐targeting peptide vaccine improved physical and cognitive function in aged mice by depleting CD38^+^ myeloid cells (Yu et al. 2025).

### 2.5 Alpha‐Ketoglutarate (AKG)

AKG, a TCA‐cycle intermediate, was first shown to extend lifespan in *C. elegans* (Chin et al. 2014) and later in mice (Asadi Shahmirzadi et al. 2020). By inhibiting ATP synthase activity and activating AMPK, AKG acts as a calorie restriction mimetic. Beyond this, AKG exerts pleiotropic effects: it suppresses inflammaging via IL‐10 induction, regulates gene expression through histone methylation, controls collagen synthesis, and preserves stem cell pluripotency (Wang et al. 2020; Bayliak and Lushchak 2021). AKG also helps maintain redox homeostasis; mechanisms include stimulating mild ROS production and thus hormesis via inhibiting ATP synthase activity, directly reacting with H_2_O_2_, and participating in the dioxygenase‐catalyzed conversion of Fe^2+^ to Fe^3+^ (Salminen et al. 2015; Bayliak and Lushchak 2021). The ongoing double‐blind ABLE trial is evaluating calcium AKG for effects on DNA‐methylation age (Sandalova et al. 2023), and AKG is now available as a dietary supplement.

### 2.6 Challenges for Metabolism Modulating Strategies

Most metabolism‐modulating strategies converge on a shared core network involving AMPK activation, NAD^+^ elevation, sirtuin activation, mTOR inhibition, and autophagy induction. Although different compounds target distinct nodes, these pathways are tightly interconnected, so perturbing one affects the others, producing shared pleiotropic effects.

Clinical translation of metabolism‐modulating strategies has produced heterogeneous outcomes, indicating strong context dependence. Whether metabolically healthy individuals benefit remains unclear, as excessive modulation may disrupt homeostasis and cause harm, exemplified by the blunted hepatic proteomic response to nutrition (le Couteur et al. 2021). Tissue specificity further complicates efficacy: mTOR inhibition is protective in adipose tissue but induces muscle dystrophy in skeletal muscle (Bentzinger et al. 2008; Polak et al. 2008).

A deeper understanding of age‐related metabolic changes will enable more effective anti‐aging strategies and clarify when CRMs are beneficial. The concept of “metabolic elasticity” describes the capacity to adapt to and recover from metabolic stress, which declines with aging and obesity and may drive metabolic deterioration (Bennett and Sato 2023; Zhou et al. 2023). Consistently, AMPK responsiveness to metabolic stimuli decreases with age (Salminen and Kaarniranta 2012). More recently, “bioenergetic capacity” has been proposed to denote the ability to maintain energy homeostasis under pathological conditions (Arnold et al. 2025). Preserving metabolic flexibility and bioenergetic capacity may therefore represent a promising metabolism‐based anti‐aging strategy.

## 3 Anti‐Oxidative and Anti‐Inflammaging Strategies

Coined by Franceschi in 2000, inflammaging refers to the immune alterations resulting from lifelong stress (Franceschi et al. 2000; Fulop et al. 2023). This phenomenon is driven by a synergistic interplay between two primary factors: the age‐related involution of the immune system (immunosenescence) and a progressive increase in pro‐inflammatory status. The latter is fueled by the senescence‐associated secretory phenotype (SASP), mitochondrial dysfunction, compromised gut barrier integrity, and the accumulation of damage‐associated molecular patterns (DAMPs). Ultimately, inflammaging manifests as a state of chronic, sterile, and low‐grade systemic inflammation. Inflammaging accelerates the development of age‐related pathologies, as advances in geosciences suggest that multiple hallmarks of aging—including stress responses, epigenetic changes, macromolecular damage, metabolic dysfunction, impaired proteostasis, and stem cell exhaustion—converge on inflammatory pathways to drive age‐related diseases (Franceschi et al. 2018). Accordingly, inflammaging is a major risk factor for age‐related morbidity and mortality (Dugan et al. 2023; Fulop et al. 2023), and reducing inflammation promotes healthy aging in preclinical models (Youm et al. 2013).

### 3.1 Mechanisms Involved in Inflammaging

**FIGURE 3:**
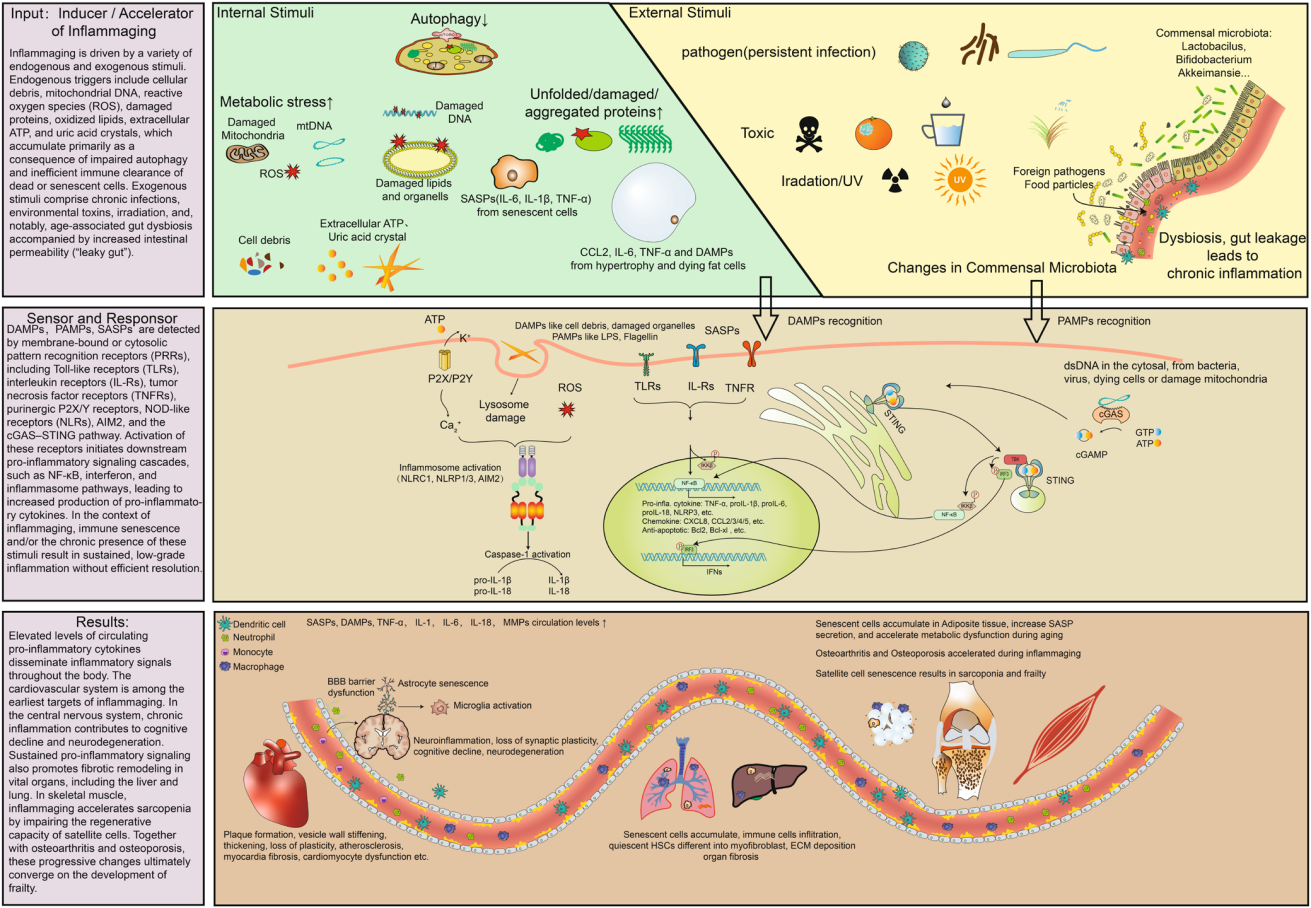
Overview of inflammaging. The upper panel depicts the potential inducers and accelerators of inflammaging; the middle panel illustrates the key receptors, signaling pathways, and downstream effectors involved; and the lower panel summarizes the detrimental consequences of inflammaging at both the cellular and organismal levels.

The machinery of inflammaging follows a bow‐tie architecture in which diverse upstream inputs converge on a small set of sensors and signaling hubs to generate broad inflammatory outputs (Figure 3). These inputs include cellular senescence and SASP factors (e.g., IL‐6, IL‐8, MMPs), DAMPs (e.g., extracellular ATP, mtDNA, misfolded proteins), PAMPs (e.g., LPS), and environmental stressors such as toxins and radiation. Furthermore, the age‐associated reactivation of retrotransposons—such as long interspersed nuclear elements (LINE‐1) and endogenous retroviruses (ERVs)—triggers innate immune sensing pathways, thereby exacerbating chronic inflammaging (de Cecco et al. 2019; Liu, Liu, et al. 2023). With aging, all of these increase due to senescent cell accumulation, gut barrier dysfunction and microbiome dysbiosis, and impaired mitochondrial function and autophagy. These stimuli are integrated by a limited group of sensors, including TLRs, cGAS, NLRs, and AHR, which activate NF‐κB, cGAS–STING, and the NLRP inflammasome to drive the production of pro‐inflammatory mediators such as IL‐1, IL‐6, IL‐18, TNF‐α, and type I interferons. Concurrently, immunosenescence reshapes immune cell composition and function, weakening host defense while sustaining chronic, low‐grade sterile inflammation (Singh et al. 2024).

### 3.2 Strategies Targeting Primary Lymphoid Involution

Functional decline in primary lymphoid organs, particularly the bone marrow and thymus, is a major driver of immunosenescence. Consequently, strategies prioritizing the preservation or reversal of thymic and bone marrow involution have emerged as pivotal anti‐aging interventions. A Phase I clinical trial evaluated the effects of recombinant human growth hormone (rhGH) in regenerating the thymus and decelerating epigenetic aging, yielding promising results (Fahy et al. 2019). The study observed an increased thymus fat‐free fraction and improved circulating monocyte profiles. Furthermore, the engraftment of young thymic epithelial cells represents another prospective strategy to rejuvenate the involuted thymus, with C‐reactive protein (CRP) levels showing favorable changes (Kim et al. 2015). A Phase II clinical study of this thymic regeneration strategy is currently underway (NCT04375657).

### 3.3 Repurposed Drugs Targeting Inflammaging

Several clinically approved drugs are being repurposed to target inflammaging in order to slow aging and prevent age‐related diseases, including the classical anti‐inflammatory drug aspirin and the mTOR inhibitor rapamycin. In addition to its CRM effects discussed in Section 2.3.3, rapamycin is well recognized for its immunosuppressive activity. By inhibiting mTOR signaling, rapamycin suppresses IL‐2–dependent T cell proliferation, thereby exerting potent immunosuppressive effects (Mukherjee and Mukherjee 2009).

Aspirin exerts anti‐inflammatory effects primarily by inhibiting COX and suppressing NF‐κB activation (Kopp and Ghosh 1994; Vane and Botting 2003). It extends lifespan in male mice (Strong et al. 2008), and long‐term use (> 60 days/year) is associated with lower frailty and faster walking speed in older men (Orkaby et al. 2021; Orkaby et al. 2022). However, aspirin use is not significantly associated with inflammatory markers such as CRP, TNFR2, or IL‐6 (Gewurz et al. 2024), suggesting that its potential anti‐aging benefits may not be mediated by suppression of chronic low‐grade inflammation.

Beyond its anti‐inflammatory actions, aspirin and its metabolite salicylate activate AMPK by binding the β subunit (Hawley et al. 2012) and inhibit mitochondrial complex I (Sandoval‐Acuña et al. 2012). They are also recognized as CRMs, as they inhibit the acetyltransferase EP300 by competing with coenzyme A, inducing global protein deacetylation and autophagy (Pietrocola et al. 2018). However, large randomized trials have dampened enthusiasm for aspirin as an anti‐aging intervention: ASPREE, ASCEND, and ARRIVE found no clinical benefit but increased bleeding risk (Gaziano et al. 2018; McNeil, Nelson, et al. 2018; McNeil, Wolfe, et al. 2018; McNeil, Woods, et al. 2018; Parish et al. 2022).

### 3.4 Targeted Anti‐Inflammatory Strategies for Anti‐Aging Strategies

The NF‐κB pathway and inflammasome activation are key drivers of inflammaging (Youm et al. 2013). Agents targeting key nodes such as IL‐1, IL‐6, IL‐11, and NLRP3, originally developed for infectious and inflammatory diseases, are now being investigated for their potential to combat inflammaging. The CANTOS trial showed that IL‐1 blockade reduces cardiovascular events and cancer incidence in patients with atherosclerosis, demonstrating that modulating inflammation can benefit age‐related disease (Ridker, MacFadyen, et al. 2017). Likewise, colchicine, a non‐selective NLRP3 inhibitor, lowers cardiovascular events in chronic coronary disease (Nidorf et al. 2020), and IL‐11–targeting antibodies extend healthspan and lifespan in preclinical models (Widjaja et al. 2024). Together, these results have intensified interest in targeting inflammation to counteract age‐related inflammaging.

Therapeutic antibodies and small molecules that block pro‐inflammatory cytokine signaling are widely used for autoimmune disorders, and many are under investigation for age‐related inflammatory diseases. Examples include Anakinra and Rilonacept (IL‐1), Tocilizumab, Siltuximab, and Sarilumab (IL‐6), and Certolizumab pegol and Infliximab (TNF‐α). Several NLRP3 inflammasome inhibitors, such as Dapansutrile, DFV890, and NT‐0796, are also in clinical development for inflammatory and age‐related conditions (Table 2).

**TABLE 2:** Anti‐inflammatory strategies in clinical development for aging and age‐related diseases.

Treatment	Status
Targeting NLRP3 inflammasome	
NT‐0796	In phase Ib/IIa trial, NT‐0796 was well tolerated and reduced neuroinflammatory biomarkers in Parkinson's disease patients (Clarke et al. 2025) Phase I study in obese participants at risk of cardiovascular disease (NCT06129409) Phase II study in obese participants with/without type 2 diabetes (NCT07055516)
OLT1177 (Dapansutrile)	Phase I safety and PK study finished (Wohlford et al. 2020) Phase II study in type 2 diabetic participants is ongoing (NCT06047262) Phase II study in the treatment of acute gout flare is ongoing (NCT05658575)
DFV890	Safety, tolerability and pharmacokinetic profile validated (Gatlik et al. 2024) Phase Ib/II study on inflammatory related diseases like coronary heart disease (NCT06031844), myeloid diseases (NCT05552469), and Osteoarthritis (NCT04886258) are ongoing or finished
Colchicine	Clinically proved capable of decrease the morbidity of secondary cardiovascular disease in patients with stable coronary artery disease (Nidorf et al. 2020)
Targeting IL‐1 pathway	
Canakinumab	Clinically proved to decrease incidence of cancer and cardiovascular events in patients with atherosclerotic diseases (Ridker, Everett, et al. 2017)
Anakinra	Clinical used to treat rheumatoid arthritis. Preclinical evaluation to cope with age related cardiovascular diseases (Ikonomidis et al. 2008; Sagris et al. 2021)
Targeting IL‐6 pathway	
Ziltivekimab	Phase II RESCUE study demonstrated that ziltivekimab is safe, and significantly reduced biomarkers of inflammation and thrombosis (Ridker et al. 2021) Phase III clinical (HERMES, ATHENA, and ZEUS) studies are ongoing to test the effects of ziltivekimab on several cardiac and cardiovascular diseases

### 3.5 Anti‐Oxidative and Anti‐Inflammatory Natural Compounds

**FIGURE 4:**
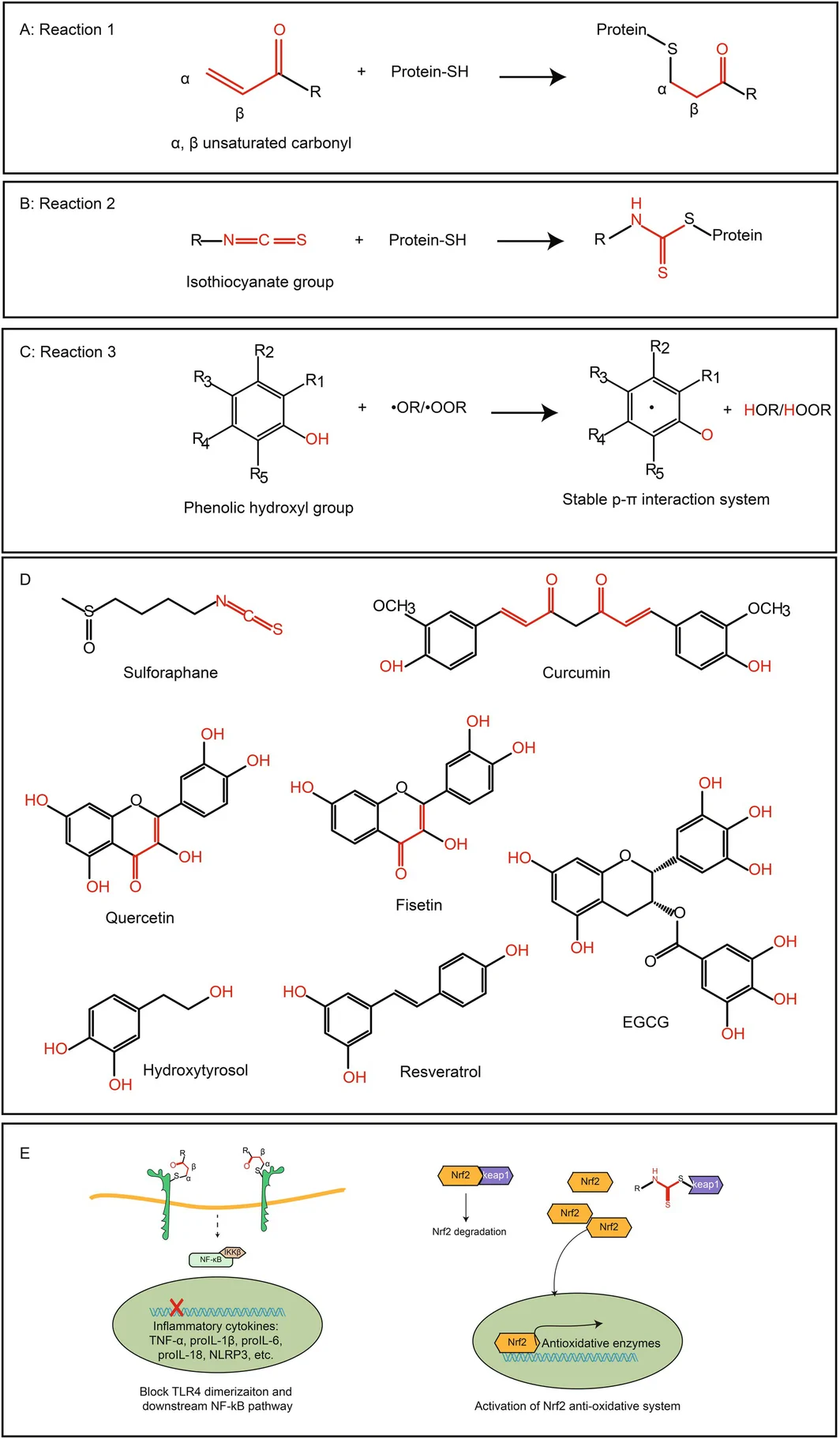
Functional moieties and mechanisms of bioactive natural products in anti‐aging interventions. Functional groups in bioactive natural products include (A) α, β‐unsaturated carbonyl groups, (B) isothiocyanate groups, and (C) phenolic hydroxyl groups. Michael addition reactions occur between compounds containing α, β‐unsaturated carbonyl or isothiocyanate groups and cysteine residues of target proteins. Compounds with phenolic hydroxyl groups exert antioxidant activity by scavenging reactive oxygen species (ROS), donating hydrogen atoms, and forming stable phenoxy radicals via p–π conjugation (C). Several well‐characterized natural products under investigation as anti‐aging candidates, along with their corresponding functional moieties, are summarized in (D). The primary mechanisms of action of these compounds include inhibition of NF‐κB signaling, activation of the Nrf2 antioxidant pathway, and direct ROS scavenging (E).

A wide range of naturally derived compounds promote healthy aging in preclinical models through anti‐oxidative and anti‐inflammatory effects (Mohammad‐Beigi et al. 2019; Zhou et al. 2019; di Rosa et al. 2020; Ellison‐Hughes 2020; Zhang et al. 2021). Representative examples include quercetin, fisetin, resveratrol, cinnamaldehyde, curcumin, vitamins C and E, omega‐3 fatty acids, hydroxytyrosol (olive oil), and epigallocatechin‐3‐gallate (EGCG, green tea) (Figure 4). Mechanistically, they act via multiple pathways: scavenging reactive oxygen species (ROS), inhibiting NF‐κB signaling, and activating the Nrf2 antioxidant system (Nani et al. 2021; Zhang et al. 2021). Compounds with α, β‐unsaturated carbonyl or isothiocyanate groups can suppress NF‐κB by interfering with cysteine‐mediated TLR4 dimerization and stabilize Nrf2 by modifying cysteine 151 in Keap1. Hydrogen bonding between berberine and NEK7 has been shown to disrupt the NEK7–NLRP3 interaction, thereby inhibiting downstream IL‐1β release (Zeng et al. 2021). Additionally, phenolic hydroxyl groups confer direct ROS‐scavenging activity, and some natural products enhance antioxidant defenses through metal chelation (Shin et al. 2020).

Most compounds in this category face major challenges for drug development, including low bioavailability, poor stability, and limited selectivity. As a result, they are often regarded as non‐pharmacological interventions and developed as nutraceuticals or phytochemicals (Dugan et al. 2023). However, these limitations may be misinterpreted. In vivo, certain compounds like phenolic acids are converted into more potent metabolites to elicit the observed activity (Heleno et al. 2015). Moreover, low serum concentrations do not necessarily equate to poor bioavailability, since some compounds may display tissue‐specific sequestering or rapid distribution into peripheral tissues following administrations (Tan et al. 2013; Chu et al. 2015).

### 3.6 Gut Microbiota

The gut microbiome and its metabolites offer a promising approach to reduce age‐related inflammation and promote healthy aging. Aging is associated with microbiome dysbiosis, reduced metabolic activity, and weakened host–microbe interactions, coinciding with increased systemic inflammation (Shin et al. 2021; Best et al. 2025). An aged gut microbiota increases the production of phenylacetic acid and phenylacetylglutamine (PAGln), which accelerate cellular senescence in mice (Yang, Wang, et al. 2025). In contrast, strains like *Akkermansia muciniphila* and *Faecalibacterium prausnitzii* mitigate inflammation by strengthening the gut barrier and producing beneficial metabolites, including short‐chain fatty acids (acetate, propionate, butyrate) and secondary bile acids such as ursodeoxycholic and lithocholic acid (Singh et al. 2024).

Implanting beneficial microbial strains or administering their metabolites holds promise for anti‐aging interventions. Urolithin A, a microbial metabolite, promotes healthy aging by activating mitophagy (Luan et al. 2021), reducing oxidative stress (Su et al. 2024), and inhibiting NF‐κB signaling and inflammation (He et al. 2024; Chen et al. 2025). Clinical trials have demonstrated that urolithin A supplementation improves muscle performance and attenuates systemic inflammation in both healthy overweight middle‐aged adults and young male athletes undergoing resistance training (Singh et al. 2022; Zhao, Zhu, et al. 2024). Another microbial metabolite, pyrroloquinoline quinone (PQQ), a vitamin‐like redox cofactor, interacts with Keap1 to disrupt Keap1–Nrf2 binding, thereby reducing oxidative stress, cellular senescence, and SASPs production, delaying age‐related intervertebral disc degeneration in mice (Xue et al. 2024). Small clinical trials also report cognitive improvements with PQQ in healthy individuals (Shiojima et al. 2022) and those with mild cognitive decline (Baltic et al. 2024). PQQ is now marketed as a dietary supplement in countries including the United States, Japan, and China.

### 3.7 Perspective of Inflammaging Targeting Strategies

Compared with CR and CRMs, inflammaging is a relatively new concept, and inflammation‐targeted anti‐aging strategies are still in their infancy. Approaches that reduce the drivers or “fuel” of inflammaging—such as antioxidants and senotherapies (see next section)—appear safer and more promising than directly manipulating inflammatory pathways. Targeting inflammation in otherwise healthy individuals requires caution, given its essential role in homeostasis. Future research to distinguish mechanisms of inflammaging from acute inflammation will be critical for developing safer, more specific interventions. A recent example is the WSTF protein, which is selectively involved in inflammaging but not acute inflammation (Wang et al. 2025).

Although the link between inflammaging, aging, and disease is well recognized in developed countries, a key question remains: does reducing inflammation actively drive the benefits of anti‐aging interventions, or is it merely a biomarker of successful strategies (Singh et al. 2024). Some argue that inflammaging is not inevitable, especially in regions with high infectious disease burden or limited food availability (McDade 2023). Studies of centenarians suggest that immune adaptability and the ability to remodel in response to harmful stimuli may be more important for healthy aging than simply suppressing inflammation (Santoro et al. 2016). Maintaining a dynamic balance between pro‐ and anti‐inflammatory cytokines appears critical for longevity (Monti et al. 2017).

## 4 Senotherapy

The accumulation of senescent cells and the resulting senescence‐associated secretory phenotype (SASP) is a major driver of age‐related inflammation. In 2011, Baker et al. showed that eliminating p16^INK4a^‐positive senescent cells delayed age‐associated phenotypes and extended both lifespan and healthspan in mice (Baker et al. 2011; Baker et al. 2016). This landmark finding has spurred intensive research into pharmacological and genetic strategies to selectively remove senescent cells, now regarded as one of the most promising anti‐aging approaches.

### 4.1 Dasatinib + Quercetin

Dasatinib and quercetin (D + Q) were the first reported senolytics (Zhu et al. 2015), which are now under active clinical investigation for several senescence‐associated diseases. Intermediate doses of D + Q have been shown to be well tolerated (Nambiar et al. 2023). In a pilot study of 14 idiopathic pulmonary fibrosis patients, D + Q significantly improved physical function (Justice et al. 2019). Short‐term D + Q treatment (11 days) in diabetic kidney disease reduced senescent cells in adipose tissue and skin, as well as circulating SASP factors, including IL‐1α, IL‐6, MMP9, and MMP12 (Hickson et al. 2019; Ellison‐Hughes 2020). Ongoing trials continue to assess its therapeutic potential (see Table 3).

**TABLE 3:** Senolytic agents under clinical development for aging and age‐related diseases.

Treatment	Status
Dasatinib + Quercetin	Accomplished studies: Safety and tolerability proved in IPF patients (Justice et al. 2019; Nambiar et al. 2023). Phase I pilot study proved capable to reduce adipose tissue senescent cell burden as well as circulating SAPSs levels include IL‐1α，IL‐6, MMP‐9 and MMP‐12 in diabetic kidney disease patients (Hickson et al. 2019). Ongoing studies: Phase I/II studies concerning safety, tolerability and efficacy to remove senescent cells in mild cognitive impairment AD patients, older adults with AD risk (NCT04785300, NCT04063124, NCT04685590, NCT05422885), and in adult survivor of childhood cancer (NCT04733534) Phase II study to evaluate its ability to improve motor, cognitive and immune functions for aging adults (NCT07000734) Phase II study for improving physical and cellular senescence elimination in frail or prefrail HIV patients (NCT07144293) Phase II study for the effect in improve skeletal health or improve osteoporosis (NCT04313634, NCT06018467) Phase II study for the effect to slow down accelerated aging in patients with mental disorders (NCT05838560) Phase I/II study for the effect in treating liver fibrosis in NASH patients (NCT05506488)
Fisetin	Accomplished study: Fisetin supplement reduced plasma IL‐8, CRP, MMP‐7 levels in patients with colorectal cancer (Farsad‐Naeimi et al. 2018) Ongoing studies: Phase I/II pilot trial study of Fisetin metabolism and effect senescent cells, control chronic inflammation in multimorbidity older individuals (NCT06431932) Phase I/II study for the efficacy in improving vascular endothelial function and reducing aortic stiffness in older individuals (NCT06133634) Phase II study of Fisetin safety and effectiveness in removing senescent cells and improve frailty in adult survivors of childhood cancer (NCT04733534); Phase II study for Fisetin efficacy in reducing senescent cells and improve frailty in various tissues of patients with peripheral artery disease (NCT06399809) Phase II study for Fisetin efficacy in reducing SASP, improve frailty and Insulin resistance in older individuals (NCT03675724)
UBX1325 (Bcl‐xl inhibitor)	UBX1325 is safe and well tolerated in diabetic macular edema (Crespo‐Garcia et al. 2024; Klier et al. 2025) Phase II clinical trial proved UBX1325 to improved vison significantly in patients with neovascular age‐related macular degeneration (NCT05275205)

### 4.2 Quercetin Analogues

The success of D + Q in eliminating senescent cells has spurred the search for more potent agents with similar mechanisms. Quercetin is a natural flavonoid with well‐established antioxidant and anti‐inflammatory effects. As noted in Section 3.4, many natural products with these properties are being explored for anti‐aging, and several also show senolytic activity. Among them, fisetin is the most studied: preclinical studies demonstrate it reduces senescent cell burden, extends lifespan, and alleviates age‐related diseases (Zhu et al. 2017; Yousefzadeh et al. 2018). Ongoing clinical trials are assessing its safety and efficacy in clearing senescent cells and reducing circulating inflammatory factors (see Table 3).

### 4.3 Dasatinib Analogues

Dasatinib, a broad‐spectrum tyrosine kinase inhibitor (TKI), selectively induces apoptosis in malignant cells dependent on tyrosine kinase signaling and in certain senescent cells that rely on these pathways to evade death (Kirkland and Tchkonia 2017; Hickson et al. 2019). Based on this, UNITY Biotechnology developed senolytics including UBX0101, UBX1325, and UBX1967. UBX0101, targeting the p53–MDM2 interaction, was the first to enter clinical testing but was discontinued due to lack of meaningful benefit (Dolgin 2020). UBX1325 and UBX1967 target Bcl‐xL; while UBX1967 has not reached clinical trials, UBX1325 has been tested in diabetic retinopathy. A single intravitreal injection of UBX1325 was safe and well tolerated in phase 1 and 2 trials, showing trends toward efficacy in diabetic macular edema over 48 weeks (Crespo‐Garcia et al. 2024; Klier et al. 2025).

Navitoclax, a Bcl‐2/Bcl‐xL/Bcl‐w inhibitor, shows potent senolytic activity but is limited by dose‐dependent thrombocytopenia because platelets depend on Bcl‐xL for survival (Zhu et al. 2016). To address this, He et al. developed the PROTAC PZ15227, which recruits Bcl‐xL to CRBN; because platelets express little CRBN, platelet toxicity is largely spared while senolytic activity is retained (He et al. 2020). Another dual PROTAC, 753b, targeting BCL‐xL and BCL‐2, selectively reduced senescent cells in aged mouse liver (Yang, Jn‐Simon, et al. 2025), although further studies are needed to establish its therapeutic potential and safety.

### 4.4 Rationally Designed Senotherapy

Beyond repurposed anti‐cancer drugs, several rationally designed senolytics have been developed. Cai et al. exploited elevated lysosomal β‐galactosidase (β‐gal) in senescent cells to create the prodrug SSK1, which is activated by β‐gal to eliminate senescent cells, reducing senescent burden, systemic inflammation, and physical decline in aged mice (Cai et al. 2020). Using a similar approach, TPE‐Gal is hydrolyzed by β‐gal to release TPE‐OH, increasing ROS and inducing senescent‐cell apoptosis; it alleviated bone loss and improved bone healing in SAMP6 mice (Gao, Hu, et al. 2025).

Small peptides have emerged as promising senolytics. An early example is the cell‐permeable FOXO4‐DRI peptide, which disrupts FOXO4–p53 interactions, releasing p53 to trigger senescent‐cell–specific apoptosis; in doxorubicin‐treated mice it alleviated frailty, alopecia, and renal dysfunction (Baar et al. 2017). A chimeric uPAR‐targeting peptide that labels senescent cells with polyglutamic acid to promote immune clearance was effective in models of liver fibrosis, lung injury, cancer, and aging (Ming et al. 2025). In addition, TAT‐PEP targeting the TBK1–ATAD3A–PINK1 axis induced senescent‐cell death in vitro (He et al. 2025). Beyond systemic applications, anti‐aging peptides have also been explored for skin rejuvenation. For example, OS‐01 reduces senescent burden, inflammation, and aging markers and is now marketed by OneSkin as a topical anti‐senescence product (Zonari et al. 2024; Zonari, Brace, Buhrer, et al. 2025; Zonari, Brace, Li, et al. 2025).

### 4.5 Senomorphics

Besides eliminating senescent cells, senomorphics aim to suppress SASPs secretion while preserving cell viability. Metformin and rapamycin were reported to reduce SASPs by inhibiting mTOR and protein synthesis (Laberge et al. 2015; Chaib et al. 2022). Similar effects are seen with glucocorticoids (corticosterone and cortisol), the JAK inhibitor ruxolitinib, NF‐κB and p38 inhibitors, and pyrroloquinoline quinone (PQQ) (Laberge et al. 2012; Xu et al. 2015; Jiang et al. 2025). Unlike intermittently dosed senolytics, senomorphics generally require continuous administration. Currently, many natural products with senolytic or senomorphic activity are being explored as anti‐aging interventions (Imb et al. 2024; Wang, Liu, et al. 2024; Gao, Yi, et al. 2025).

### 4.6 Safety Concerns of Senotherpy

While senotherapy holds significant therapeutic promise, its clinical application is tempered by critical safety concerns. Primary among these are specific toxicities, such as thrombocytopenia associated with BCL‐X_L‐targeting senolytics (Sharma et al. 2020), the potential risk of compromising senescence‐mediated tumor suppression (Schmitt 2017), and the finding that senescent cells play a beneficial role in limiting liver fibrosis (Krizhanovsky et al. 2008). Consistent with this, Grosse et al. demonstrated that the majority of p16‐high senescent cells in aged mice are vascular endothelial cells located in liver sinusoids; the systemic elimination of these cells can trigger hepatic and perivascular fibrosis, leading to overall health deterioration (Grosse et al. 2020). Furthermore, a recent study highlighted the essential role of senescent cells in maintaining blood–brain barrier integrity, underscoring the need for diligence when deploying serotherapeutic strategies (Watson et al. 2026). Consequently, rigorous preclinical and clinical evaluations are mandatory to address these safety risks before senotherapy can be translated into clinical practice.

## 5 Stem Cell‐Based Therapy

Tissue‐specific stem cells switch from quiescence to proliferation in response to injury to replace damaged cells, maintaining tissue homeostasis and delaying age‐related decline (Sameri et al. 2020). With aging, this regenerative capacity wanes due to stem cell exhaustion or reduced responsiveness. Consequently, maintaining stem cell potency or stem cell replacement has been proposed to counteract organ aging, and stem cell therapies for conditions such as osteoporosis and neurodegenerative diseases are under active investigation.

### 5.1 Mesenchymal Stem Cells (MSCs)

MSCs are leading candidates for anti‐aging therapies. These multipotent cells, found in most adult tissues, are readily isolated and can differentiate into osteocytes, adipocytes, chondrocytes, and fibroblasts (Pittenger et al. 1999). MSCs also exert anti‐inflammatory and immunomodulatory effects and, due to low MHC/HLA class I and absence of class II expression, evade immune recognition, enhancing their persistence and therapeutic efficacy (Ryan et al. 2005; Lee et al. 2009).

MSCs have been extensively investigated for promoting bone regeneration, treating osteoporosis, reducing senescent cell burden, and alleviating frailty during aging (Lin et al. 2019; He et al. 2021; Zhu et al. 2021; Wang, Deng, et al. 2024). Their therapeutic potential has also been explored in age‐related diseases such as diabetes and neurodegenerative disorders. Clinical studies indicate that both autologous and allogeneic MSCs exhibit favorable safety and tolerability profiles (see Table 4). Evidence of primary efficacy has been reported in patients with diabetes (Wu et al. 2024), Parkinson's disease (Zhuo et al. 2024), and age‐related frailty (Tompkins et al. 2017), with improvements in immune function observed in several trials. Beyond these clinical applications, a recent study showed that intravenous administration of genetically modified, senescence‐resistant MSCs markedly slowed aging in aged macaques (Lei et al. 2025). Collectively, these findings highlight the substantial promise of MSCs—including genetically enhanced MSCs—as a powerful anti‐aging strategy.

**TABLE 4:** Clinical trials concerning MSCs.

Stem cells	Condition	Outcome
Autologous MSC	Type I diabetic patients	Transplantation is safe, and significantly reduced the number of hypoglycemic episodes (Izadi et al. 2022)
MSC + MC (mononuclear cells)	Type II diabetic patients	MSC and MC therapy reduced the incidence of chronic diabetes complications and improved metabolic control of T2D patients (Wu et al. 2024)
Autologous MSC	ALS	Phase I clinical trials proved the safety of MSC transplantation into the central nervous system (Mazzini et al. 2012)
MSC secreting Neurotrophic factor (MSC‐NTFs)	ALS	MSC‐NTFs transplantation was safe and well tolerated, and further clinical trials were required to confirm the possibility of clinical benefits (Petrou et al. 2016)
MSC‐NTFs	ALS	In this phase III trial, MSC‐NTFs transplantation fails to generate statistically significant improvement in response rate, but notable improvements in cerebrospinal biomarkers of neuroinflammation, neurodegeneration, and neurotrophic factors were observed (Cudkowicz et al. 2022)
Hypoxia‐preconditioned olfactory mucosa‐MSC (hOM‐MSC)	PD	Phase I study proved that intraspinal transplantation of hOM‐MSCs improved the recovery of neurologic function while regulated the neuroinflammatory response in PD patients (Zhuo et al. 2024)
Allogeneic MSCs	Aging frailty	Phase I study proved the safety profile of intravenous infusion of allogeneic MSCs. Improvement in mobility and immunological status were also observed (Golpanian et al. 2017) Phase II study also showed improved physical performance and immunological status (Tompkins et al. 2017)
Allogeneic MSCs	Diabetic kidney disease	Allogeneic MSCs were safe and well‐tolerated in diabetic kidney disease patients (Perico et al. 2023)

### 5.2 Maintain MSCs Potency

Strategies prioritizing the preservation of endogenous mesenchymal stem cell (MSC) potency represent a promising regenerative frontier. Beyond micronutrients and phytochemicals that shield MSCs from oxidative stress, non‐psychotropic cannabidiol and extracellular matrix (ECM) components—such as tropoelastin and hyaluronan—have proven capable of maintaining MSC stemness (Wong et al. 2017; Lee et al. 2024; Kot et al. 2025; Chueaphromsri et al. 2026). Furthermore, targeting mitochondrial homeostasis via Nrf1 overexpression effectively mitigates age‐related MSC senescence (Lee et al. 2025).

### 5.3 Stem Cells Derived Extracellular Vesicles

A major limitation of MSC‐based anti‐aging strategies is the difficulty in ensuring both the quantity and quality of therapeutic cells. Allogeneic MSCs exhibit donor‐to‐donor biological variability, whereas autologous MSCs are often limited in number, particularly in individuals with systemic diseases. In addition, safety concerns—especially the potential tumorigenicity of MSCs—and uncertainties regarding long‐term efficacy require further clinical validation.

To address these challenges, MSC‐derived exosomes and other extracellular vesicles (EVs), including microvesicles (MVs), are being explored as cell‐free alternatives that recapitulate many of the therapeutic effects of MSC transplantation (He et al. 2021). EVs from human embryonic stem cells or young stem cells can rejuvenate senescent stem cells and delay age‐related degeneration both in vitro and in vivo (Cai et al. 2024; Bi et al. 2025). Similarly, MSC‐derived EVs exert senoprotective effects in mouse models of osteoarthritis and in ex vivo–cultured human end‐stage osteoarthritic cartilage (Boulestreau et al. 2024; Feng et al. 2024). Moreover, Liu et al. showed that embedding MSC‐derived MVs into an ROS‐responsive hydrogel (Hydrogel@iMVs) produced robust therapeutic effects in a mouse model of age‐related osteoarthritis (Liu, Cheng, et al. 2023).

## 6 Parabiosis

Parabiosis—the experimental connection of the circulatory systems of two individuals—was proved to have rejuvenating effects toward the older individual (Ludwig and Elashoff 1972; Conboy et al. 2005), inspiring searches for rejuvenating factors in young serum, or identification of detrimental factors in old serum. A complex network of systemic mediators—including hormones, metabolites, cytokines, extracellular vesicles, and miRNAs—coordinates inter‐organ communication to dictate metabolic homeostasis and aging (Tokizane and Imai 2025). The most well‐studied factors are Chemokine CCL11, which is identified as a detrimental factors (Villeda et al. 2011), and GDF11, that is the most promising identified rejuvenating factor among others (Loffredo et al. 2013; Katsimpardi et al. 2014; Sinha et al. 2014; Katsimpardi et al. 2020). A pharmaceutical company, Elevian, is founded to translate GDF11 from research to the clinic. A clinical trial investigating the use of GDF11 for stroke treatment is currently under consideration.

Beyond soluble factors, extracellular vesicles and mitochondria function as mobile signals propagating systemic aging cues. Adipose‐secreted eNAMPT, for instance, maintains NAD^+^ homeostasis across distant organs via the bloodstream (Yoon et al. 2015; Yoshida et al. 2019). This inter‐organ axis has paved the way for EVs and mitochondrial transfer therapies, an emerging anti‐aging frontier aimed at restoring cellular energetics by replacing senescent organelles (Zhao, Dong, et al. 2024; Du et al. 2026).

## 7 Epigenetic Rejuvenation

DNA methylation has transitioned from a passive hallmark of aging to a superior mortality predictor (DNAmAge) and a reversible mediator of biological decline. Modulating DNAm status—via DNMT inhibitors 5‐Aza‐dC or natural compounds such as dihydromyricetin and theobromine—can rescue age‐related visual decline and rejuvenate skin (Chen et al. 2020; Falckenhayn et al. 2023; Saad et al. 2025). Furthermore, methylation‐supportive lifestyle interventions and α‐ketoglutarate‐based formulations (e.g., Rejuvant) have demonstrated DNAmAge reductions of 3–8 years, effectively decoupling chronological age from biological health (Demidenko et al. 2021; Fitzgerald et al. 2021).

Beyond the induction of pluripotency, the anti‐aging field is shifting toward partial reprogramming to reverse cellular senescence. Ectopic OSK expression (Oct4, Sox2, and Klf4) has been shown to restore youthful DNA methylation landscapes, thereby promoting axonal regeneration and reversing vision loss in aged or glaucomatous mouse models (Lu et al. 2020). This paradigm has transitioned to clinical translation with the FDA‐approved Phase I trial of ER‐100, marking the first human endeavor to rejuvenate ocular cells via cellular reprogramming. Collectively, these milestones establish epigenetic rejuvenation as a transformative frontier for decoupling chronological age from biological decline.

## 8 Future Directions

The field of anti‐aging research has advanced rapidly over the past decade. Metabolism‐modulating interventions—including exercise, CR, CRMs, and bioactive phytochemicals—represent major therapeutic avenues. In parallel, senotherapy has emerged as one of the most promising strategies to target fundamental aging mechanisms. Meanwhile, stem cell–based therapies and the targeted modulation of inflammaging are developing as additional frontiers in anti‐aging research.

The current development pipeline for anti‐aging interventions remains immature, as reflected by the high failure rate in translating experimental strategies into clinical applications. Major obstacles include the limited validation of tractable molecular targets and the lack of standardized screening platforms. Most existing screening systems rely on senescence‐based cellular assays, typically using replicatively aged or DNA damage–induced senescent fibroblasts to identify compounds that selectively eliminate senescent cells. Other cell‐based models assess mitochondrial dysfunction, oxidative stress, or mTORC1/AMPK activity to screen for compounds that modulate metabolism and stress responses. However, the extent to which these in vitro systems faithfully recapitulate human aging remains unclear.

Secondly, the routine use of animal models to evaluate drug efficacy may be neither appropriate nor necessary for developing anti‐aging interventions. “New Approach Methodologies” (NAMs)—including computational modeling, advanced in vitro assays, and organ‐on‐a‐chip systems based on human‐derived materials or data—may offer greater relevance, as emphasized in the FDA's *Roadmap to Reducing Animal Testing in Preclinical Safety Studies* (Food, U.S. and A. Drug 2025). The integrated application of large‐scale data analysis, AI‐driven modeling, and human‐derived organoids, organ‐on‐chip platforms, or other microphysiological systems could substantially improve the identification of robust targets and biomarkers of human aging, thereby accelerating the development of effective anti‐aging strategies.

Thirdly, clinical evaluation of anti‐aging interventions remains poorly standardized, both in study design and in the selection of efficacy biomarkers. Most trials are currently conducted in the context of specific age‐related diseases, using disease‐related biomarkers to assess outcomes. Commonly measured indicators include circulating inflammatory factors such as IL‐6, CRP, TNF‐α, and MMPs. With the advancement of large‐scale data analysis and AI‐based modeling, molecular biomarkers of aging—such as DNA methylation clocks and transcriptomic aging signatures—may provide more robust and informative tools to bridge preclinical findings with clinical outcomes.

Looking ahead, future screening platforms are expected to integrate multi‐scale experimental systems with high‐content phenotyping, single‐cell omics, and AI‐driven predictive modeling to enable more efficient and physiologically relevant discovery of anti‐aging interventions.

## Author Contributions

Song Huang led the project. Song Huang and Feifei Li developed the conceptual framework, Feifei Li conducted the comprehensive literature review, and drafted the contents for each section of the manuscript. Yankai Wang provided substantial input on the mechanism of inflammaging and anti‐inflammaging strategies. Gelin Wang and Jiguo Chen offered critical feedback on the texts and figures and were involved in revising the manuscript. All authors contributed to the editing and final review of the manuscript.

## Funding

This work was supported by the National Health Commission of the People's Republic of China. Ministry of Science and Technology of the People's Republic of China.

## Conflicts of Interest

The authors declare no conflicts of interest.

## Data Availability

Data sharing not applicable to this article as no datasets were generated or analysed during the current study.
